# Association Between Dietary Magnesium Intake and Low Muscle Mass: The Mediating Role of Inflammatory Indicators †

**DOI:** 10.3390/healthcare14010001

**Published:** 2025-12-19

**Authors:** Zhu Zhu, Wenji Wang, Feng Ding, Yue Shen

**Affiliations:** 1 Department of Geriatrics, Shanghai Ninth People’s Hospital, Shanghai Jiao Tong University School of Medicine, Shanghai 200127, China; pearlyzz@163.com; 2 Department of Nephrology, Shanghai Ninth People’s Hospital, Shanghai Jiao Tong University School of Medicine, Shanghai 200127, China; wangwj1736@sh9hospital.org.cn

**Keywords:** sarcopenia, dietary magnesium, novel inflammatory indicators, NHANES, cross-sectional analysis

## Abstract

**Background:** Magnesium is essential for mitochondrial function and muscle regeneration, potentially protecting against low muscle mass (LMM). We examined the association of dietary magnesium intake with LMM risk and skeletal muscle index (SMI), and whether inflammatory indicators mediate this relationship. **Methods:** A total of 5793 participants aged 20–59 years were included in this study from the National Health and Nutrition Examination Survey (NHANES) conducted from 2011 to 2018. To investigate the association of dietary magnesium intake with LMM and SMI, we applied weighted logistic regression model, linear regression model, restricted cubic spline analysis, subgroup analysis and sensitivity analysis. Inflammatory indicators were assessed using mediation analysis, including the C-reactive protein–albumin–lymphocyte (CALLY) index, neutrophil–platelet score (NP), platelet-to-albumin ratio (PAR) and red blood cell distribution width-to-albumin ratio (RAR) mediation. **Results:** In the fully adjusted model, participants in the highest magnesium quartile had a reduced risk of LMM, with OR of 0.33 (95% CI: 0.18, 0.60), and increased levels of SMI, with β values of 0.05 (95% CI: 0.04, 0.07). Mediation analysis showed that NP, PAR, and RAR mediated 18%, 13%, and 21% of the association between magnesium and LMM, respectively, and also acted as mediators of the relationship between magnesium and SMI, with mediation ratios of 32%, 24%, and 25%, respectively. **Conclusion:** Higher dietary magnesium intake was associated with lower LMM risk and higher SMI, partly mediated through inflammatory indicators involving NP, PAR, and RAR. This finding may provide a new perspective on the prevention and management of LMM.

## 1. Introduction

Sarcopenia is a chronic, systemic musculoskeletal disorder characterized by a decline in muscle mass, strength, and function, which is closely associated with a range of adverse outcomes, including falls, frailty, diminished functional capacity, and increased mortality [1,2,3]. Low muscle mass (LMM) is a key characteristic of sarcopenia. Basic research indicates that elevated oxidative stress contributes to muscle atrophy through protein degradation, suppressed synthesis, and mitochondrial dysfunction [4,5].

Magnesium is an essential mineral involved in critical physiological processes at the cellular level, widely present in foods such as beef, whole grains, nuts, and leafy greens [6,7]. It promotes adenosine triphosphate (ATP) production to support muscle contraction and protein synthesis, and delays the progression of LMM by suppressing the kappa-B (NF-κB) pathway to reduce inflammatory mediators like TNF-α and IL-6 [8,9,10]. While serum magnesium is a common clinical measure, it only decreases in cases of severe deficiency; dietary intake better reflects long-term nutritional status [11].

Inflammatory markers, including neutrophils, lymphocytes, platelets, and C-reactive protein (CRP), interact with cytokines like IL-1, IL-6, and TNF-α, influencing systemic inflammation [12,13,14]. Composite indices such as the C-reactive protein–albumin–lymphocyte (CALLY) index, neutrophil–platelet score (NP), platelet-to-albumin ratio (PAR) and red blood cell distribution width-to-albumin ratio (RAR) provide more robust assessments of inflammation, nutrition, and immune status than single markers, and have predictive value in conditions including infections and cancer [15,16,17,18]. We therefore selected these four indices as potential mediators to explore whether inflammatory pathways underlie the association between dietary magnesium and LMM.

Epidemiological studies examining dietary magnesium intake in relation to LMM and the role of inflammatory mediators remain limited. This study seeks to examine the relationship between dietary magnesium consumption and LMM and skeletal muscle index (SMI) levels, and the mediating role of inflammatory indicators in the association. Our goal is to explore nutritional factors associated with LMM to provide insights for future nutritional prevention and control strategies.

## 2. Materials and Methods

### 2.1. Study Population

The NHANES is a comprehensive, stratified, multi-stage survey aimed at assessing the health and nutritional status of U.S. adults and children. NHANES has been approved by the National Center for Health Statistics (NCHS) Ethics Review Board (ERB) with written consent from all participants.

**Figure 1:**
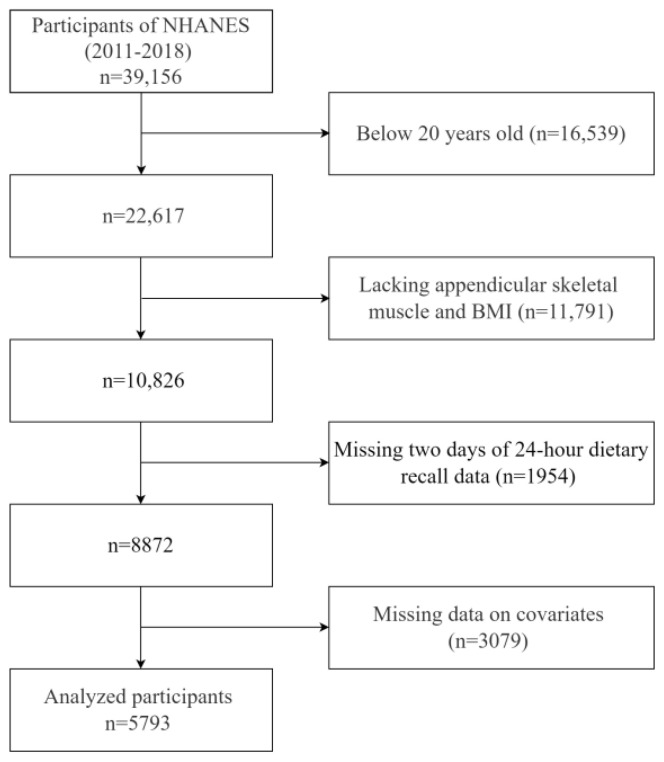
Flowchart of participant selection from NHANES 2011–2018. **Notes:** Abbreviation: NHANES, National Health and Nutrition Examination Survey.

For this study, we used data collected over 4 NHANES cycles, 2011–2012, 2013–2014, 2015–2016, and 2017–2018, from 39,156 individuals. Our study excluded specific populations as outlined in Figure 1: (1) aged  <  20 years (n = 16,539); (2) without appendicular skeletal muscle and BMI data (n = 11,791); (3) missing two days of 24 h dietary recall data (n = 1954); (4) lacking covariates data (n = 3079). Ultimately, 5793 participants aged 20–59 years were enrolled in the final analysis.

### 2.2. Assessment of Dietary Nutrient Intake

The NHANES collected nutritional data through two 24 h dietary assessments. An initial in-person interview was performed at Mobile Examination Center (MEC), followed by a telephone recall 3–10 days subsequently. For our analysis, we derived magnesium consumption values by averaging both dietary reports, while total intake included dietary and supplementary magnesium.

### 2.3. Inflammatory Indicators

The Coulter DxH 800 analyzer (Beckman Coulter, Shanghai, China) was employed to measure lymphocyte, neutrophil, platelet counts, and red cell distribution width (RDW) from all participants’ venous blood samples in the study. The SYNCHRON System’s High Sensitivity C-Reactive Protein (CRP) reagent employs the highly sensitive near-infrared Particle Immunoassay rate methodology. Albumin concentration is measured using the dye bromcresol purple (BCP) as the method. Particularly, CRP values were only available for the sample of NHANES 2015–2018, and analysis of the CALLY index is restricted to this time period.

Detailed laboratory methods are provided on the NHANES website, and indicators of inflammation are calculated using the following equations:

CALLY index = Albumin (g/dL) × Lymphocyte (/μL)/CRP (mg/dL)

NP = Neutrophil (/μL) × Platelet score (/μL)

PAR = Platelet (/μL)/Albumin (g/dL)

RAR = RDW (%)/Albumin (g/dL)

### 2.4. LMM and Skeletal Muscle Index

Dual-energy X-ray absorptiometry (DXA) scanning was conducted to measure muscle mass in the arms and legs, facilitating the assessment of skeletal muscle mass. Individuals who were pregnant, or weighed over 136 kg or exceeded 196 cm in height, were excluded from the DXA assessments [19]. Due to the absence of DXA data for individuals over 60 years old in the NHANES, there is a gap in muscle mass data for the elderly population. The skeletal muscle index (SMI) was calculated as the appendicular skeletal muscle mass divided by body mass index (BMI). LMM was diagnosed according to the criterion by the Foundation for the National Institutes of Health (FNIH), specifically SMI values <0.512 for women and <0.789 for men [20].

### 2.5. Covariates

The covariates adjusted for this study were as follows: age, race, gender, poverty–income ratio (PIR), education level, smoking history, drinking status, levels of physical activity, energy and protein intake, history of diabetes and hypertension, estimated glomerular filtration rate [(eGFR), mL/min per 1.73 m^2^] and serum 25-hydroxyvitamin D [25(OH)D_3_, nmol/L]. We adjusted for these covariates to estimate the direct effect of magnesium intake on LMM.

The poverty-to-income ratio (PIR) values were categorized into three groups: below 1.39, 1.39 to 3.49, and above 3.49. Individuals classified as smokers have a history of smoking over 100 cigarettes in their lifetime. Drinking status is categorized as never, former and current. In terms of physical activity, participants were divided into inactive and active categories based on the World Health Organization 2020 guidelines on physical activity and sedentary behavior [21]. Hypertension was defined as a systolic blood pressure (SBP) ≥ 140 mmHg, a diastolic blood pressure (DBP) ≥ 90 mmHg, or a self-reported history of a hypertension diagnosis by a healthcare provider [22]. Diabetes was diagnosed when fasting plasma glucose (FPG) reached 7.0 mmol/L or higher, or random blood glucose levels was 11 mmol/L or higher, HbA1c was 6.5% or greater, or the use of oral hypoglycemic agents, or insulin injections, or the individual had a previous diagnosis of diabetes reported by a doctor [23]. Chronic kidney disease (CKD) was diagnosed based on an estimated glomerular filtration rate (eGFR) below 60 mL/min per 1.73 m^2^, or a urine albumin-to-creatinine ratio (ACR) of 30 mg/g or higher [24].

### 2.6. Statistical Analysis

Our analyses incorporated NHANES’s multi-stage sampling methodology by adjusting for clusters, stratification, and weighted sampling using the R package “survey” (v 4.4-8). Continuous variables were expressed as mean ± standard or medians (interquartile ranges) and compared by *t*-tests or Mann–Whitney U test depending on whether they followed a normal distribution. Categorical variables were expressed as N(%) and tested using chi-square tests.

Magnesium intake, inflammatory markers and SMI were ln-transformed to improve the skewed distribution. We adhered to the principle of simplicity, starting with the most basic model to test the primary hypotheses. Weighted logistic and weighted linear regression were utilized to assess the relationships of magnesium with LMM and SMI. Magnesium was included in the model as continuous variables and quartiles (Q1, 2.25–5.40; Q2, 5.41–5.70; Q3, 5.71–5.99; Q4, 6.00–7.69), with the first quartile serving as the reference group. Three models were constructed. Model 1 was unadjusted. Model 2 adjusted for age, gender, and race. Model 3 adjusted for age, race, gender, PIR, education level, smoking history, drinking status, levels of physical activity, energy and protein intake, history of diabetes and hypertension, eGFR and 25(OH)D_3_. The dose–response relationships between magnesium with LMM and SMI were explored utilizing restricted cubic splines models with three knots based on the fully adjusted model. Subgroup analyses were performed on the basis of gender, age, education, hypertension, diabetes, drinking history, and chronic kidney disease (CKD). The 187 participants with extreme energy intake (males consuming <800 or >4200 kcal/day; females <500 or >3500 kcal/day) were further excluded, and the main analyses were performed to ensure the robustness of the results. To investigate whether inflammatory indicators mediated the relationship between dietary magnesium and LMM and SMI, we utilized the R package “mediator” and performed 1000 bootstrap simulations to estimate the mediating effect of each mediator and calculate the proportion of mediators. For ease of interpretation, all mediation effects presented in the report are unstandardized coefficients.

Statistical analysis was conducted using R Studio (version 4.2.2), with a significance threshold of *p*-value < 0.05 (two-tailed).

## 3. Results

### 3.1. Baseline Characteristics of the Study

A total of 5793 participants were enrolled in the study, with a median age of 38 years, and 51.77% were male. A total of 416 (7.18%) of the participants were diagnosed with LMM. Compared to those without LMM, participants with LMM were more likely to be older, Mexican American or other Hispanic, had lesser educational attainment, a lower PIR, reduced activity levels, had history of hypertension and diabetes, lower intake of energy and protein and lower serum 25(OH)D_3_ level. For inflammatory markers, significant differences were shown in participants with and without LMM. Individuals diagnosed with LMM exhibited reduced CALLY level alongside elevated NP, PAR, and RAR levels. The characteristics of the participants are presented in Table 1.

### 3.2. Associations Between Dietary Magnesium Intake with LMM

Table 2 presents the associations between dietary magnesium intake and LMM. The continuous magnesium showed a consistent negative association with LMM across all models. In Model 3, the risk of LMM decreased by 68% for each unit increment in magnesium. When magnesium was categorized, participants in the third and fourth quartiles exhibited a negative correlation with LMM relative to the reference group. In Model 3, participants in the third and fourth quartiles had a reduced risk of LMM, with ORs of 0.63 (95% CI: 0.41, 0.96) and 0.33 (95% CI: 0.18, 0.60), respectively.

### 3.3. Associations Between Dietary Magnesium Intake with SMI

As shown in Table 3, the continuous magnesium was positively associated with SMI in all models. In Model 3, each unit increase in magnesium was related to an increase in SMI [β: 0.05; 95% CI: (0.03, 0.06)]. Compared with the first quartile of magnesium, the third and fourth quartiles of magnesium were positively correlated with SMI in all models. In Model 3, magnesium levels in the third and fourth quartiles were associated with increased SMI levels, with β values of 0.03 (95% CI: 0.01, 0.05) and 0.05 (95% CI: 0.04, 0.07), respectively. Sensitivity analysis confirmed the robustness of these findings, showing consistent results after excluding participants with extreme energy intake (Tables S1 and S2).

### 3.4. Dose–Response Relationship

**Figure 2:**
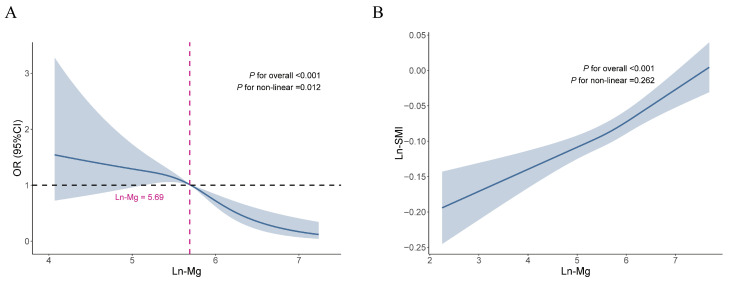
Application of restricted cubic spline regression to examine the association between magnesium and (**A**) LMM and (**B**) SMI. **Notes:** Abbreviation: LMM, low muscle mass; SMI, skeletal muscle index. Adjusted for age, race, gender, PIR, education level, smoking history, drinking status, levels of physical activity, energy and protein intake, history of diabetes and hypertension, eGFR and 25(OH)D_3_.

The restricted cubic spline model was constructed to explore the potential nonlinear association between magnesium and LMM and SMI levels based on Model 3 (Figure 2). The model showed a nonlinear association between magnesium and LMM, with an inflection point of 5.69 (*P*_nonlinear_ = 0.012). The association with LMM was not significant until magnesium reached the inflection point (Table S3). When magnesium exceeded the inflection point, there was a significant protective effect against LMM, with OR of 0.06 (95% CI: 0.02, 0.19). The nonlinear correlation between magnesium and SMI was not significant (*P*_nonlinear_ = 0.262).

### 3.5. Subgroup Analysis

**Figure 3:**
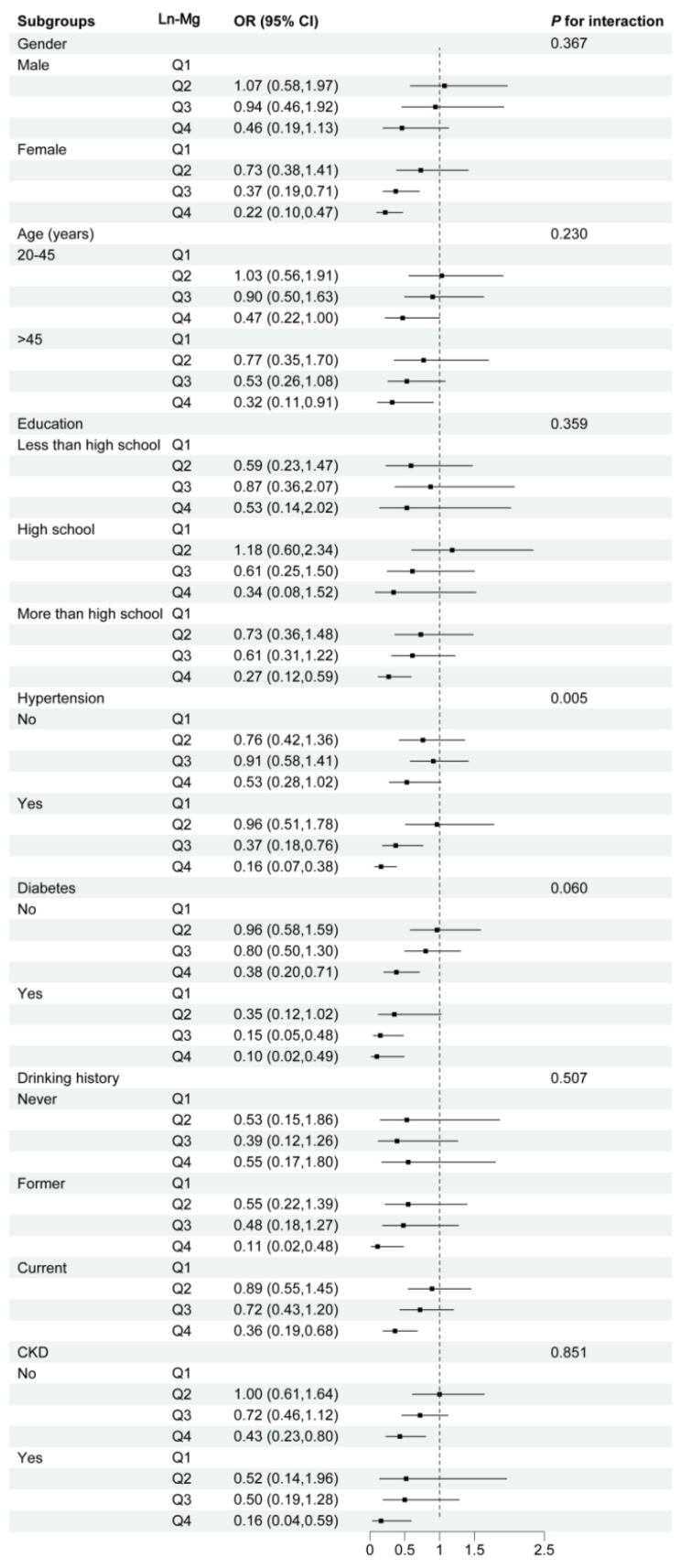
Subgroup analysis for the association between magnesium and LMM. **Notes:** Abbreviation: LMM, low muscle mass; CKD, chronic kidney disease. Adjusted for age, race, gender, PIR, education level, smoking history, drinking status, levels of physical activity, energy and protein intake, history of diabetes and hypertension, eGFR and 25(OH)D_3_.

In subgroup analysis, a significant interaction was observed between magnesium and history of hypertension in the association between magnesium and LMM (*P* for interaction = 0.005). The protective effect of magnesium against LMM was significant only in participants with hypertension. The risk of LMM was significantly lower in hypertensive individuals with magnesium in the third and fourth quartiles compared with the first quartile, with ORs of 0.37 (95% CI: 0.18, 0.76) and 0.16 (95% CI: 0.07, 0.38), respectively (Figure 3). The protective effect of magnesium against LMM was more pronounced in females and participants over 45 years and with a history of diabetes and CKD, compared to males, 20–45 years of age, and without history of diabetes and CKD. Similarly, we observed a positive correlation between dietary magnesium and SMI in most subgroups (Figure S1). However, no significant interactions were observed in these subgroups.

### 3.6. Associations Between Dietary Mg Intake with Inflammatory Indicators

Whether continuous or quartiles, there was a consistent positive correlation between magnesium and CALLY in all models (Table 4). In contrast, the association between magnesium and NP, PAR, and RAR was negative. In Model 3, magnesium in the fourth quartile was significantly positively correlated with CALLY compared with the lowest quartile [β: 0.35; 95% CI: (0.18, 0.53], and negatively correlated with NP, PAR, and RAR, with β of −0.12 (95% CI: −0.17, −0.07), −0.05 (95% CI: −0.08, −0.03) and −0.04 (95% CI: −0.05, −0.03), respectively.

### 3.7. Associations Between Inflammatory Indicators with LMM and SMI

The association between CALLY and LMM was negative, while the association between NP, PAR and RAR and LMM was positive (Table 5). In Model 3, the risk of LMM decreased by 45% for every one-unit increment in CALLY. With the elevated levels of NP, PAR, and RAR, the increased risk of LMM, with ORs of 2.46 (95% CI: 1.96, 3.09), 4.07 (95% CI: 2.68, 6.19) and 2.27 (95% CI: 1.83, 2.81), respectively, we observed a positive correlation between CALLY and SMI levels, and a negative correlation between NP, PAR, RAR and SMI levels (Table 6).

### 3.8. Mediating Role of Inflammatory Indicators

**Figure 4:**
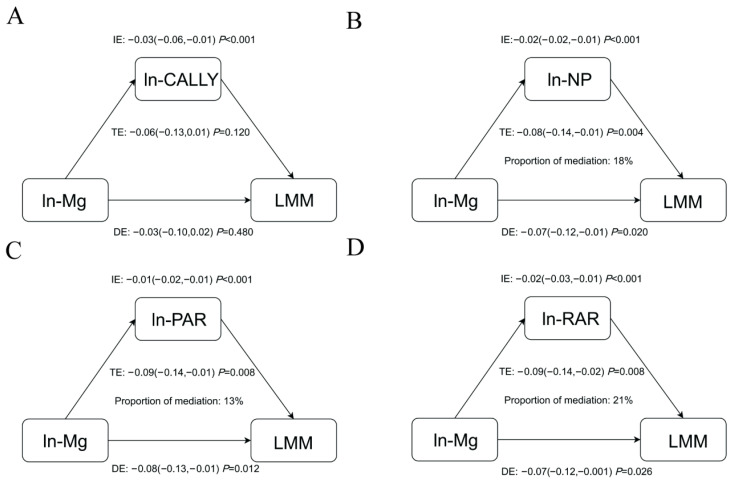
The mediation effects of inflammatory indicators ((**A**) CALLY index, (**B**) NP, (**C**) PAR, (**D**) RAR) on the association of dietary magnesium intake and LMM. **Notes:** Abbreviations: LMM, low muscle mass; CALLY, C-reactive protein–albumin–lymphocyte; NP, neutrophil–platelet score; PAR, platelet-to-albumin ratio; RAR, red blood cell distribution width-to-albumin ratio. Adjusted for age, race, gender, PIR, education level, smoking history, drinking status, levels of physical activity, energy and protein intake, history of diabetes and hypertension, eGFR and 25(OH)D_3_.

**Figure 5:**
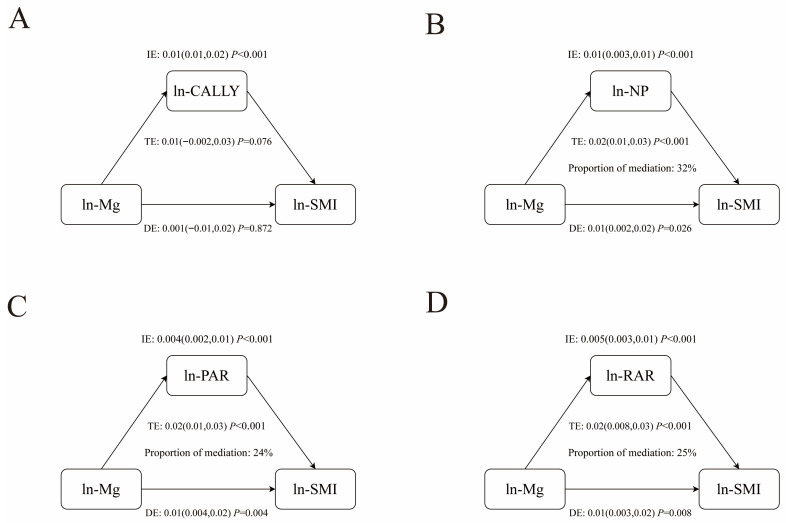
The mediation effects of inflammatory indicators ((**A**) CALLY index, (**B**) NP, (**C**) PAR, (**D**) RAR) on the association of dietary magnesium intake and SMI. **Notes:** Abbreviations: CALLY, C-reactive protein–albumin–lymphocyte; NP, neutrophil–platelet score; PAR, platelet-to-albumin ratio; RAR, red blood cell distribution width-to-albumin ratio; SMI, skeletal muscle index. Adjusted for age, race, gender, PIR, education level, smoking history, drinking status, levels of physical activity, energy and protein intake, history of diabetes and hypertension, eGFR and 25(OH)D_3_.

Given the intercorrelations between magnesium, inflammatory markers (CALLY index, NP, PAR, RAR) and LMM and SMI, further mediated effects analyses were performed (Figure 4 and Figure 5). NP, PAR, and RAR mediated 18%, 13%, and 21% of the association between magnesium and LMM, respectively (Table 7). NP, PAR, and RAR also acted as mediators of the association between magnesium and SMI, with 32%, 24%, and 25% of mediation, respectively (Table 8). However, no significant mediating effect of CALLY in the association between magnesium and LMM and SMI was observed.

## 4. Discussion

In this cross-sectional study, we examined the relationship between dietary magnesium consumption and LMM and SMI levels, while exploring the mediating effect of inflammatory indicators. Our study focused on the middle-aged population aged 20–59 to investigate the risk factors for LMM to help identify individuals at high risk for developing severe sarcopenia at an earlier life stage, enabling timely lifestyle intervention recommendations. The findings indicate that higher magnesium consumption through diet is associated with lower LMM risk and increased SMI levels. Meanwhile, the associations between dietary magnesium intake and LMM and SMI were partially mediated by NP, RAR, and RAR.

Magnesium serves as a vital nutrient for maintaining human health, contributing significantly to disease prevention and therapeutic interventions across various conditions [25,26,27,28]. Restricted cubic spline results revealed that there was a dose–response relationship between magnesium and LMM, and the protective effect on LMM was significant when ln-Mg reached 5.69 mg/d (equivalent to 298.87 mg/d of Mg). The Food and Nutrition Board (FNB) at the Institute of Medicine established age- and gender-specific magnesium intake guidelines, known as the Recommended Dietary Allowance (RDA). For the U.S. adult population, these recommendations specify daily magnesium requirements of 400 mg for men aged 19–30 years, increasing to 420 mg for those over 30. Women’s RDAs are set at 310 mg for ages 19–30 and 320 mg for women beyond 30 years of age [29]. Our results show that the actual daily magnesium intake of U.S. adults is lower than the recommended amount. Therefore, as suggested by the RDA, maintaining an adequate magnesium intake through the daily diet is beneficial and may have the potential function of preventing LMM and improving SMI levels.

The results of several studies were similar to ours. The InCHIANTI Aging Study including 1138 older adults revealed significant associations between magnesium levels and multiple measures of physical performance, including grip strength, calf muscle strength, knee extension torque, and ankle extension isometric strength [30]. A cross-sectional analysis of 776 participants revealed lower magnesium intake was associated with higher hs-CRP and lower muscle mass [31]. Animal models showed that magnesium supplementation promoted the anabolic effect on muscle function and morphology by inhibiting fat accumulation and muscle fiber protein degradation and alleviated corticosteroid-associated muscle atrophy in rats [32].

One of the major factors inducing LMM is mitochondrial dysfunction [33]. Magnesium serves as a vital regulator in cellular physiology. Within mitochondria, Mg^2+^ ions modulate protein biosynthesis, ATP production, and multiple enzymatic reactions, while insufficient intracellular magnesium concentrations trigger oxidative damage and impair mitochondrial performance [34,35,36]. At the same time, magnesium depletion leads to an increase in the production of reactive oxygen species (ROS), which induces the activation of NF-κB and decreases the expression of MyoD, thereby inhibiting myogenesis [37,38].

Notably, in the associations between dietary magnesium and inflammatory markers, as well as between inflammatory markers and LMM and SMI, CALLY index exhibited an opposite directionality compared to other inflammatory markers. This may be due to the higher CALLY index indicating better nutritional status and lower inflammation levels. Adequate magnesium intake maintains lymphocyte proliferation function, promotes protein synthesis, improves nutritional status, reduces inflammatory factor levels, and facilitates the migration and proliferation of muscle stem cells [39,40].

Mediation analysis revealed that NP, PAR, and RAR partially mediated the relationship between dietary magnesium and LMM risk and SMI levels, indicating that magnesium intake may maintain muscle function by modulating these novel inflammatory indicators. Inadequate magnesium intake may elevate platelet reactivity, increase neutrophil infiltration, and reduce albumin synthesis, leading to elevated NP and PAR. This thrombotic inflammatory state impairs skeletal muscle microcirculation, disrupts energy and nutrient supply to muscles, and causes muscle ischemia, hypoxia, and inflammatory damage [41,42,43]. Magnesium deficiency may reduce red blood cell count, accelerate erythrocyte aging, and increase reticulocyte count, elevating RAR levels. Consequently, muscle tissue cannot obtain sufficient nutritional and oxygen support, leading to atrophy and functional loss [44,45].

Subgroup analyses showed an interaction between dietary magnesium and LMM in the hypertensive subgroup, which significantly reduced the risk of LMM in hypertensive patients. This may be due to the fact that hypertensive patients exhibit increased oxidative stress and often in a chronic low-grade inflammatory state, and inflammation promotes muscle protein catabolism and increases the risk of LMM [46,47]. Dietary magnesium may help maintain muscle mass by reducing the inflammatory response and decreasing muscle protein breakdown. There were no significant interactions between dietary magnesium and LMM and SMI levels across variables such as age, gender, and education, suggesting that this protective effect of dietary magnesium can be generalized to U.S. populations with different characteristics. Sensitivity analysis confirmed the robustness of these findings, showing consistent results after excluding participants with extreme energy intake.

Our study has several strengths, including the first exploration of the association of dietary magnesium with LMM and SMI levels in a representative U.S. population sample, as well as the mediating effects of inflammatory markers including the CALLY index, NP, PAR, and RAR, with subgroup analyses and sensitivity analyses further supporting our findings. However, the cross-sectional nature of NHANES limited our ability to infer causality. In addition, we used data from interviews, such as self-reported dietary intake, which may be biased by recall and reporting. Furthermore, the absence of DXA data for adults aged 60 years and older in the database limits the generalizability of our findings to this clinically crucial demographic. Future studies should utilize nationally representative cohorts that include the full adult age spectrum and employ longitudinal designs or randomized controlled trials to establish temporality, clarify potential reverse causality, and validate our observations.

## 5. Conclusions

This study highlights the associations between dietary magnesium intake and LMM risk and SMI levels, and reveals the mediating role of novel inflammatory markers (CALLY index, NP, PAR, RAR) in the association.

## Figures and Tables

**Table 1:** Baseline characteristics of study participants by incident LMM.

Characteristics	Total (n = 5793)	Controls (n = 5377)	LMM (n = 416)	*p*
**Age (years)**	38.00 (28.00, 48.00)	38.00 (28.00, 48.00)	44.00 (32.00, 54.00)	<0.001
**Gender, N(%)**				0.499
Male	2999 (51.77)	2777 (51.65)	222 (53.37)	
Female	2794 (48.23)	2600 (48.35)	194 (46.63)	
**Race, N(%)**				<0.001
Mexican American	791 (13.65)	641 (11.92)	150 (36.06)	
Other Hispanic	517 (8.92)	463 (8.61)	54 (12.98)	
Non-Hispanic white	2285 (39.44)	2164 (40.25)	121 (29.09)	
Non-Hispanic black	1171 (20.21)	1142 (21.24)	29 (6.97)	
Others	1029 (17.76)	967 (17.98)	62 (14.90)	
**Education level, N(%)**				<0.001
Less than high school	762 (13.15)	661 (12.29)	101 (24.28)	
High school	1217 (21.01)	1093 (20.33)	124 (29.81)	
More than high school	3814 (65.84)	3623 (67.38)	191 (45.91)	
**PIR, N(%)**				<0.001
≤1.39	1780 (30.73)	1617 (30.07)	163 (39.18)	
1.39–3.49	1988 (34.32)	1832 (34.07)	156 (37.50)	
>3.49	2025 (34.96)	1928 (35.86)	97 (23.32)	
**Physical activity level, N(%)**				0.001
Low	1353 (23.36)	1229 (22.86)	124 (29.81)	
High	4440 (76.64)	4148 (77.14)	292 (70.19)	
**Smoking history, N(%)**				0.446
No	3589 (61.95)	3324 (61.82)	265 (63.70)	
Yes	2204 (38.05)	2053 (38.18)	151 (36.30)	
**Drinking status, N(%)**				<0.001
Never	620 (10.70)	557 (10.36)	63 (15.14)	
Former	612 (10.56)	543 (10.10)	69 (16.59)	
Current	4561 (78.73)	4277 (79.54)	284 (68.27)	
**History of hypertension, N(%)**				<0.001
No	4242 (73.23)	3992 (74.24)	250 (60.10)	
Yes	1551 (26.77)	1385 (25.76)	166 (39.90)	
**History of** **diabetes, N(%)**				<0.001
No	5236 (90.38)	4910 (91.31)	326 (78.37)	
Yes	557 (9.62)	467 (8.69)	90 (21.63)	
**Energy (kcal)**	2041.50 (1593.00, 2627.50)	2057.00 (1600.50, 2639.00)	1910.50 (1513.88, 2504.12)	0.001
**Protein (g)**	80.36 (60.77, 105.18)	80.65 (61.04, 105.59)	75.50 (57.94, 99.35)	0.005
**EGFR (mL/min/1.73 m^2^)**	104.31 (92.00, 116.17)	104.00 (91.76, 116.06)	107.88 (95.83, 118.45)	0.007
**25(OH)D_3_ (nmol/L)**	56.80 (40.70, 73.58)	57.50 (41.20, 74.00)	49.50 (35.48, 65.96)	<0.001
**Ln-CALLY**	5.70 (0.46)	5.70 (0.46)	5.60 (0.42)	<0.001
**Ln-NP**	2.62 (1.28)	2.69 (1.27)	1.81 (1.11)	<0.001
**Ln-PAR**	6.83 (0.51)	6.81 (0.50)	7.04 (0.51)	<0.001
**Ln-RAR**	4.01 (0.27)	4.00 (0.27)	4.09 (0.29)	<0.001
**Ln-Mg**	1.13 (0.13)	1.13 (0.13)	1.19 (0.15)	<0.001
**Ln-SMI**	−0.23 (0.25)	−0.20 (0.24)	−0.51 (0.23)	<0.001

**Notes:** *p* < 0.05 indicates a statistically significant difference. Abbreviations: LMM, low muscle mass; PIR, poverty/income ratio; EGFR, estimated glomerular filtration rate; 25(OH)D_3_, 25-hydroxyvitamin D; CALLY, C-reactive protein–albumin–lymphocyte; NP, neutrophil–platelet score; PAR, platelet-to-albumin ratio; RAR, red blood cell distribution width-to-albumin ratio; Mg, magnesium; SMI, skeletal muscle index.

**Table 2:** Association between dietary magnesium intake and LMM.

	Model 1	*p*	Model 2	*p*	Model 3	*p*
	OR (95% CI)		OR (95% CI)		OR (95% CI)	
**Mg, per 50 mg increment**	0.84 (0.80, 0.88)	<0.001	0.79 (0.75, 0.83)	<0.001	0.80 (0.76, 0.85)	<0.001
**Ln-Mg as continuous**	0.40 (0.32, 0.51)	<0.001	0.29 (0.23, 0.36)	<0.001	0.32 (0.24, 0.44)	<0.001
**Ln-Mg quartile**						
Q1	Ref		Ref		Ref	
Q2	0.86 (0.57, 1.29)	0.472	0.76 (0.49, 1.19)	0.238	0.84 (0.54, 1.31)	0.424
Q3	0.68 (0.47, 0.98)	0.044	0.52 (0.36, 0.76)	0.001	0.63 (0.41, 0.96)	0.033
Q4	0.33 (0.21, 0.52)	<0.001	0.23 (0.14, 0.36)	<0.001	0.33 (0.18, 0.60)	<0.001

**Notes:** *p* < 0.05 indicates a statistically significant difference. Model 1: unadjusted. Model 2: adjusted for age, gender, and race. Model 3: adjusted for age, race, gender, PIR, education level, smoking history, drinking status, levels of physical activity, energy and protein intake, history of diabetes and hypertension, eGFR and 25(OH)D_3_. Abbreviation: LMM, low muscle mass; Mg, magnesium.

**Table 3:** Association between dietary magnesium intake and SMI.

	Model 1	*p*	Model 2	*p*	Model 3	*p*
	β (95% CI)		β (95% CI)		β (95% CI)	
**Mg, per 50 mg increment**	0.02 (0.02, 0.02)	<0.001	0.01 (0.01, 0.01)	<0.001	0.005 (0.002, 0.01)	<0.001
**Ln-Mg as continuous**	0.16 (0.14, 0.18)	<0.001	0.08 (0.07, 0.09)	<0.001	0.05 (0.03, 0.06)	<0.001
**Ln-Mg quartile**						
Q1	Ref		Ref		Ref	
Q2	0.05 (0.02, 0.08)	<0.001	0.03 (0.01, 0.05)	0.003	0.02 (−0.0006, 0.03)	0.066
Q3	0.12 (0.09, 0.14)	<0.001	0.05 (0.04, 0.07)	<0.001	0.03 (0.01, 0.05)	<0.001
Q4	0.20 (0.18, 0.23)	<0.001	0.09 (0.08, 0.11)	<0.001	0.05 (0.04, 0.07)	<0.001

**Notes:** *p* < 0.05 indicates a statistically significant difference. Abbreviation: SMI, skeletal muscle index; Mg, magnesium. Model 1: unadjusted. Model 2: adjusted for age, gender, and race. Model 3: adjusted for age, race, gender, PIR, education level, smoking history, drinking status, levels of physical activity, energy and protein intake, history of diabetes and hypertension, eGFR and 25(OH)D_3_.

**Table 4:** Association between dietary magnesium intake and inflammation indicators (CALLY index, NP, PAR, RAR).

	Model 1	*p*	Model 2	*p*	Model 3	*p*
	β (95% CI)		β (95% CI)		β (95% CI)	
**Outcome: ln-CALLY**						
**Ln-Mg as continuous**	0.46 (0.36, 0.57)	<0.001	0.39 (0.28, 0.49)	<0.001	0.36 (0.21, 0.50)	<0.001
**Ln-Mg quartile**						
Q1	Ref		Ref		Ref	
Q2	0.22 (0.08, 0.36)	0.002	0.21 (0.08, 0.35)	0.002	0.16 (0.02, 0.30)	0.025
Q3	0.33 (0.19, 0.47)	<0.001	0.27 (0.13, 0.41)	<0.001	0.19 (0.04, 0.35)	0.013
Q4	0.54 (0.41, 0.68)	<0.001	0.45 (0.31, 0.59)	<0.001	0.35 (0.18, 0.53)	<0.001
**Outcome: ln-NP**						
**Ln-Mg as continuous**	−0.10 (−0.13, −0.07)	<0.001	−0.09 (−0.12, −0.06)	<0.001	−0.12 (−0.16, −0.08)	<0.001
**Ln-Mg quartile**						
Q1	Ref		Ref		Ref	
Q2	−0.03 (−0.07, 0.01)	0.163	−0.04 (−0.07, 0.002)	0.059	−0.04 (−0.08, −0.01)	0.049
Q3	−0.07 (−0.11, −0.03)	<0.001	−0.07 (−0.11, −0.04)	<0.001	−0.08 (−0.12, −0.04)	<0.001
Q4	−0.12 (−0.15, −0.08)	<0.001	−0.10 (−0.14, −0.06)	<0.001	−0.12 (−0.17, −0.07)	<0.001
**Outcome: ln-PAR**						
**Ln-Mg as continuous**	−0.07 (−0.09, −0.06)	<0.001	−0.03 (−0.04, −0.01)	<0.001	−0.05 (−0.07, −0.03)	<0.001
**Ln-Mg quartile**						
Q1	Ref		Ref		Ref	
Q2	−0.02 (−0.04, −0.01)	0.018	−0.01 (−0.03, 0.003)	0.141	−0.02 (−0.04, 0.001)	0.054
Q3	−0.06 (−0.08, −0.04)	<0.001	−0.03 (−0.05, −0.01)	0.003	−0.04 (−0.06, −0.02)	<0.001
Q4	−0.09 (−0.11, −0.07)	<0.001	−0.04 (−0.06, −0.02)	<0.001	−0.05 (−0.08, −0.03)	<0.001
**Outcome: ln-RAR**						
**Ln-Mg as continuous**	−0.05 (−0.06, −0.04)	<0.001	−0.02 (−0.03, −0.02)	<0.001	−0.03 (−0.04, −0.02)	<0.001
**Ln-Mg quartile**						
Q1	Ref		Ref		Ref	
Q2	−0.02 (−0.03, −0.01)	<0.001	−0.01 (−0.02, −0.01)	0.003	−0.02 (−0.03, −0.01)	<0.001
Q3	−0.04 (−0.05, −0.03)	<0.001	−0.02 (−0.03, −0.01)	<0.001	−0.03 (−0.04, −0.02)	<0.001
Q4	−0.06 (−0.07, −0.05)	<0.001	−0.03 (−0.04, −0.02)	<0.001	−0.04 (−0.05, −0.03)	<0.001

**Notes:** *p* < 0.05 indicates a statistically significant difference. Abbreviations: Mg, magnesium; CALLY, C-reactive protein–albumin–lymphocyte; NP, neutrophil–platelet score; PAR, platelet-to-albumin ratio; RAR, red blood cell distribution width-to-albumin ratio. Model 1: unadjusted. Model 2: adjusted for age, gender, and race. Model 3: adjusted for age, race, gender, PIR, education level, smoking history, drinking status, levels of physical activity, energy and protein intake, history of diabetes and hypertension, eGFR and 25(OH)D_3_.

**Table 5:** Association between inflammation indicators (CALLY index, NP, PAR, RAR) and LMM.

	Model 1	*p*	Model 2	*p*	Model 3	*p*
	OR (95% CI)		OR (95% CI)		OR (95% CI)	
**Ln-CALLY**	0.56 (0.50, 0.63)	<0.001	0.51 (0.45, 0.59)	<0.001	0.55 (0.48, 0.64)	<0.001
**Ln-NP**	2.51 (2.05, 3.08)	<0.001	2.63 (2.11, 3.27)	<0.001	2.46 (1.96, 3.09)	<0.001
**Ln-PAR**	3.46 (2.38, 5.01)	<0.001	4.61 (3.04, 6.98)	<0.001	4.07 (2.68, 6.19)	<0.001
**Ln-RAR**	2.08 (1.75, 2.46)	<0.001	2.59 (2.12, 3.17)	<0.001	2.27 (1.83, 2.81)	<0.001

**Notes:** *p* < 0.05 indicates a statistically significant difference. Abbreviations: LMM, low muscle mass; CALLY, C-reactive protein–albumin–lymphocyte; NP, neutrophil–platelet score; PAR, platelet-to-albumin ratio; RAR, red blood cell distribution width-to-albumin ratio. Model 1: unadjusted. Model 2: adjusted for age, gender, and race. Model 3: adjusted for age, race, gender, PIR, education level, smoking history, drinking status, levels of physical activity, energy and protein intake, history of diabetes and hypertension, eGFR and 25(OH)D_3_.

**Table 6:** Association between inflammation indicators (CALLY index, NP, PAR, RAR) and SMI.

	Model 1	*p*	Model 2	*p*	Model 3	*p*
	β (95% CI)		β (95% CI)		β (95% CI)	
**Ln-CALLY**	0.07 (0.07, 0.08)	<0.001	0.04 (0.04, 0.04)	<0.001	0.04 (0.03, 0.04)	<0.001
**Ln-NP**	−0.13 (−0.14, −0.12)	<0.001	−0.06 (−0.07, −0.05)	<0.001	−0.05 (−0.06, −0.05)	<0.001
**Ln-PAR**	−0.33 (−0.35, −0.31)	<0.001	−0.11 (−0.12, −0.10)	<0.001	−0.10 (−0.11, −0.08)	<0.001
**Ln-RAR**	−0.65 (−0.69, −0.60)	<0.001	−0.23 (−0.25, −0.20)	<0.001	−0.18 (−0.21, −0.16)	<0.001

**Notes:** *p* < 0.05 indicates a statistically significant difference. Abbreviations: CALLY, C-reactive protein–albumin–lymphocyte; NP, neutrophil–platelet score; PAR, platelet-to-albumin ratio; RAR, red blood cell distribution width-to-albumin ratio; SMI, skeletal muscle index. Model 1: unadjusted. Model 2: adjusted for age, gender, and race. Model 3: adjusted for age, race, gender, PIR, education level, smoking history, drinking status, levels of physical activity, energy and protein intake, history of diabetes and hypertension, eGFR and 25(OH)D_3_.

**Table 7:** The mediation effects of inflammatory indicators (CALLY index, NP, PAR, RAR) on the association of dietary magnesium intake and LMM.

Mediation	Total Effects	*p*	Indirect Effects	*p*	Direct Effects	*p*	Mediated Proportion	*p*
**Ln-CALLY**	−0.06 (−0.13, 0.01)	0.120	−0.03 (−0.06, −0.01)	<0.001	−0.03 (−0.10, 0.02)	0.480	0.54 (−2.62, 3.11)	0.120
**Ln-NP**	−0.08 (−0.14, −0.01)	0.004	−0.02 (−0.02, −0.01)	<0.001	−0.07 (−0.12, −0.01)	0.020	0.18 (0.08, 0.55)	0.004
**Ln-PAR**	−0.09 (−0.14, −0.01)	0.008	−0.01 (−0.02, −0.01)	<0.001	−0.08 (−0.13, −0.01)	0.012	0.13 (0.05, 0.39)	0.008
**Ln-RAR**	−0.09 (−0.14, −0.02)	0.008	−0.02 (−0.03, −0.01)	<0.001	−0.07 (−0.12, −0.001)	0.026	0.21 (0.11, 0.64)	0.008

**Notes:** *p* < 0.05 indicates a statistically significant difference. Abbreviations: LMM, low muscle mass; CALLY, C-reactive protein–albumin–lymphocyte; NP, neutrophil–platelet score; PAR, platelet-to-albumin ratio; RAR, red blood cell distribution width-to-albumin ratio. Adjusted for age, race, gender, PIR, education level, smoking history, drinking status, levels of physical activity, energy and protein intake, history of diabetes and hypertension, eGFR and 25(OH)D_3_.

**Table 8:** The mediation effects of inflammatory indicators (CALLY index, NP, PAR, RAR) on the association of dietary magnesium intake and SMI.

Mediation	Total Effects	*p*	Indirect Effects	*p*	Direct Effects	*p*	Mediated Proportion	*p*
**Ln-CALLY**	0.01 (−0.002, 0.03)	0.076	0.01 (0.01, 0.02)	<0.001	0.001 (−0.01, 0.02)	0.872	0.92 (−2.81, 7.56)	0.076
**Ln-NP**	0.02 (0.01, 0.03)	<0.001	0.01 (0.003, 0.01)	<0.001	0.01 (0.002, 0.02)	0.026	0.32 (0.16, 0.79)	<0.001
**Ln-PAR**	0.02 (0.01, 0.03)	<0.001	0.004 (0.002, 0.01)	<0.001	0.01 (0.004, 0.02)	0.004	0.24 (0.12, 0.53)	<0.001
**Ln-RAR**	0.02 (0.008, 0.03)	<0.001	0.005 (0.003, 0.01)	<0.001	0.01 (0.003, 0.02)	0.008	0.25 (0.14, 0.65)	<0.001

**Notes:** *p* < 0.05 indicates a statistically significant difference. Abbreviations: CALLY, C-reactive protein–albumin–lymphocyte; NP, neutrophil–platelet score; PAR, platelet-to-albumin ratio; RAR, red blood cell distribution width-to-albumin ratio; SMI, skeletal muscle index. Adjusted for age, race, gender, PIR, education level, smoking history, drinking status, levels of physical activity, energy and protein intake, history of diabetes and hypertension, eGFR and 25(OH)D_3_.

## Data Availability

The data presented in this study are available in the National Health and Nutrition Examination Survey (NHANES) repository at https://wwwn.cdc.gov/nchs/nhanes/ (accessed on 1 March 2025), reference period 2010–2018. These data were derived from publicly available resources maintained by the Centers for Disease Control and Prevention (CDC).

## Supplementary Materials

The following supporting information can be downloaded at: https://www.mdpi.com/article/10.3390/healthcare14010001/s1, Figure S1: Subgroup analysis for the association between magnesium and SMI; Table S1: Sensitive analysis between the dietary magnesium intake and LMM (excluding the participants with extreme energy intake); Table S2: Sensitive analysis between the dietary magnesium intake and SMI (excluding the participants with extreme energy intake); Table S3: The threshold and effect of magnesium on LMM.

## Abbreviations

The following abbreviations are used in this manuscript:

NHANES	National Health and Nutrition Examination Survey
LMM	low muscle mass
SMI	skeletal muscle index
CKD	chronic kidney disease
CALLY	C-reactive protein–albumin–lymphocyte
NP	neutrophil–platelet score
PAR	platelet-to-albumin ratio
RAR	red blood cell distribution width-to-albumin ratio
