# Supplementary material for: Association Between Dietary Magnesium Intake and Low Muscle Mass: The Mediating Role of Inflammatory Indicators
Source: Healthcare (Basel). 2025 Dec 19;14(1):1. doi: 10.3390/healthcare14010001 (PMC12785460; doi:10.3390/healthcare14010001)
Supplement: Supplementary file 1 [file healthcare-14-00001-s001.zip › healthcare-3920466-supplementary.pdf]

Table S1. Sensitive analysis between the dietary magnesium intake and sarcopenia (excluding the participants with extreme energy intake).

	**Model 1**		**Model 2**		**Model 3**	
	**OR (95%CI)**	***P***	**OR (95%CI)**	***P***	**OR (95%CI)**	***P***
**Ln-Mg as continuous**	0.40 (0.30, 0.52)	<0.001	0.27 (0.20, 0.35)	<0.001	0.28 (0.19, 0.40)	<0.001
**Ln-Mg quartile**						
Q1	Ref		Ref		Ref	
Q2	0.85 (0.57, 1.28)	0.441	0.75 (0.48, 1.18)	0.216	0.80 (0.51, 1.25)	0.325
Q3	0.65 (0.44, 0.96)	0.036	0.50 (0.34, 0.74)	0.001	0.58 (0.38, 0.87)	0.013
Q4	0.35 (0.22, 0.56)	<0.001	0.24 (0.15, 0.38)	<0.001	0.32 (0.18, 0.57)	<0.001

Notes:

Abbreviation: Mg, magnesium.

Model 1: unadjusted.

Model 2: adjusted for age, gender, and race.

Model 3: adjusted for age, race, gender, PIR, education level, smoking history, drinking status, levels of physical activity, energy and protein intake, history of diabetes and hypertension, eGFR and 25(OH)D3.

Table S2. Sensitive analysis between the dietary magnesium intake and SMI (excluding the participants with extreme energy intake).

	**Model 1**		**Model 2**		**Model 3**	
	**β (95%CI)**	***P***	**β (95%CI)**	***P***	**β (95%CI)**	***P***
**Ln-Mg as continuous**	0.17 (0.15, 0.18)	<0.001	0.08 (0.07, 0.09)	<0.001	0.04 (0.03, 0.06)	<0.001
**Ln-Mg quartile**						
Q1	Ref		Ref		Ref	
Q2	0.05 (0.02, 0.08)	<0.001	0.03 (0.01, 0.05)	0.005	0.02 (-0.002, 0.03)	0.081
Q3	0.12 (0.09, 0.15)	<0.001	0.05 (0.04, 0.07)	<0.001	0.03 (0.01, 0.04)	<0.001
Q4	0.20 (0.17, 0.23)	<0.001	0.09 (0.08, 0.11)	<0.001	0.05 (0.03, 0.07)	<0.001

Notes:

Abbreviation: Mg, magnesium.

Model 1: unadjusted.

Model 2: adjusted for age, gender, and race.

Model 3: adjusted for age, race, gender, PIR, education level, smoking history, drinking status, levels of physical activity, energy and protein intake, history of diabetes and hypertension, eGFR and 25(OH)D3.

Table S3. The threshold and effect of ln-Mg on sarcopenia.

**Ln-Mg**	**OR（95% CI）**	***P***
< 5.69	0.58 (0.26, 1.31)	0.184
> 5.69	0.06 (0.02, 0.19)	<0.001

Notes:

Abbreviation: Mg, magnesium.

Adjusted for age, race, gender, PIR, education level, smoking history, drinking status, levels of physical activity, energy and protein intake, history of diabetes and hypertension, eGFR and 25(OH)D3.

Subgroups	Ln-Mg	β (95% CI)	*P* for interaction
Gender			0.082
Male	Q1		
	Q2	0.002 (-0.007,0.011)	
	Q3	0.005 (-0.005,0.016)	
	Q4	0.018 (0.006,0.030)	
Female	Q1		
	Q2	0.009 (-0.002,0.020)	
	Q3	0.019 (0.008,0.029)	
	Q4	0.026 (0.015,0.037)	
Age (years)			0.266
20-45	Q1		
	Q2	0.002 (-0.007,0.010)	
	Q3	0.008 (-0.001,0.017)	
	Q4	0.017 (0.007,0.027)	
>45	Q1		
	Q2	0.012 (-0.001,0.025)	
	Q3	0.013 (0.000,0.026)	
	Q4	0.021 (0.006,0.036)	
Education			0.181
Less than high school	Q1		
	Q2	0.004 (-0.010,0.019)	
	Q3	0.008 (-0.009,0.025)	
	Q4	0.009 (-0.011,0.030)	
High school	Q1		
	Q2	0.007 (-0.007,0.021)	
	Q3	0.007 (-0.009,0.022)	
	Q4	0.017 (-0.003,0.036)	
More than high school	Q1		
	Q2	0.009 (-0.001,0.018)	
	Q3	0.017 (0.008,0.025)	
	Q4	0.028 (0.019,0.037)	
Hypertension			0.340
No	Q1		
	Q2	0.007 (-0.002,0.015)	
	Q3	0.013 (0.005,0.020)	
	Q4	0.023 (0.014,0.032)	
Yes	Q1		
	Q2	0.007 (-0.006,0.019)	
	Q3	0.013 (-0.002,0.029)	
	Q4	0.023 (0.007,0.040)	
Diabetes			0.810
No	Q1		
	Q2	0.006 (-0.001,0.014)	
	Q3	0.013 (0.006,0.020)	
	Q4	0.024 (0.016,0.031)	
Yes	Q1		
	Q2	0.013 (-0.002,0.029)	
	Q3	0.015 (-0.003,0.033)	
	Q4	0.015 (-0.007,0.036)	
Drinking history			0.883
Never	Q1		
	Q2	-0.001 (-0.019,0.018)	
	Q3	0.011 (-0.015,0.037)	
	Q4	0.020 (-0.003,0.043)	
Former	Q1		
	Q2	0.002 (-0.017,0.020)	
	Q3	0.004 (-0.015,0.023)	
	Q4	0.022 (0.002,0.041)	
Current	Q1		
	Q2	0.009 (0.000,0.017)	
	Q3	0.014 (0.007,0.022)	
	Q4	0.024 (0.015,0.033)	
CKD			0.472
No	Q1		
	Q2	0.005 (-0.002,0.012)	
	Q3	0.010 (0.003,0.017)	
	Q4	0.019 (0.011,0.027)	
Yes	Q1		
	Q2	0.006 (-0.016,0.027)	
	Q3	0.019 (0.000,0.039)	
	Q4	0.032 (0.006,0.057)	

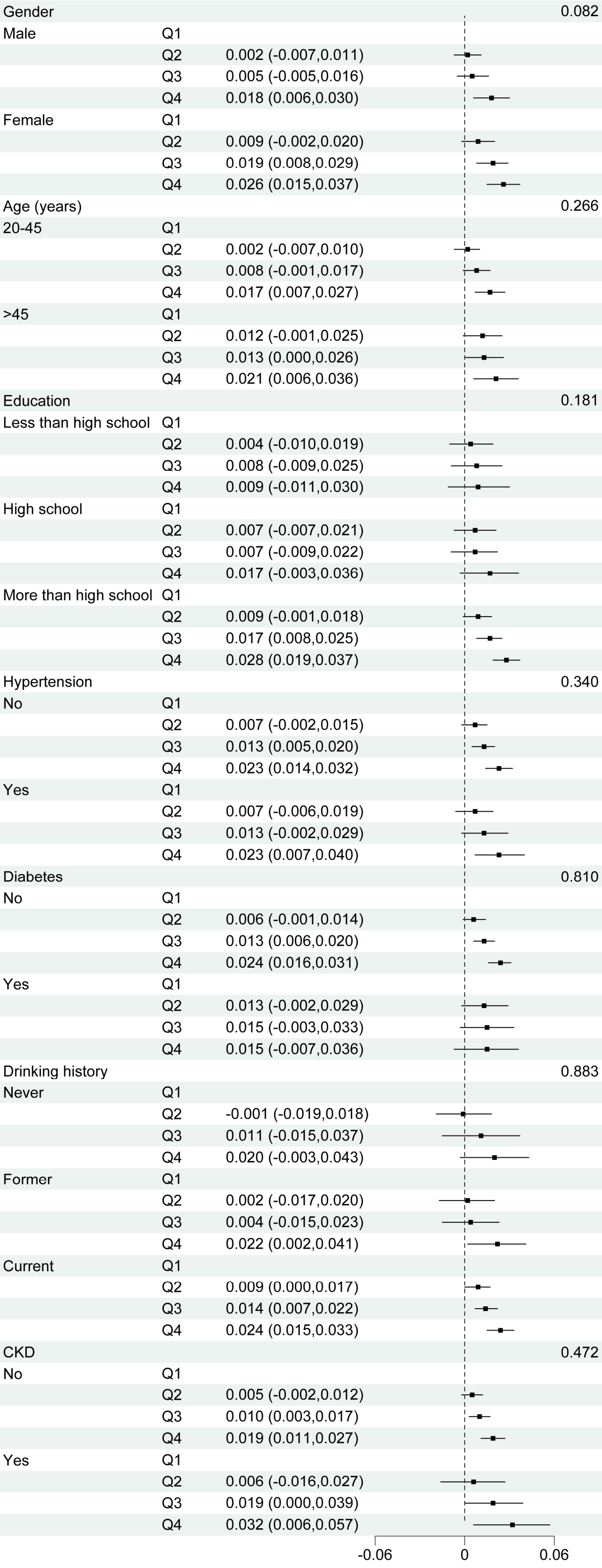

Figure S1. Subgroup analysis for the association between ln-Mg and ln-SMI.

Notes:

Abbreviation: Mg, magnesium; CKD, chronic kidney disease; SMI, skeletal muscle index.

Adjusted for age, race, gender, PIR, education level, smoking history, drinking status, levels of physical activity, energy and protein intake, history of diabetes and hypertension, eGFR and 25(OH)D3.
